# Stopping Fistula Hemorrhage without Bleeding Time and Money - A Low Cost, Low Resource Hemodialysis Fistula Model for Emergency Medicine Residents

**DOI:** 10.5070/M5.52204

**Published:** 2026-04-30

**Authors:** Mary Jordan, Thomas Yang, Michael Collier, Lacie Bailey

**Affiliations:** * Medical College of Wisconsin, Department of Emergency Medicine, Milwaukee, WI; ^ Icahn School of Medicine at Mount Sinai, Department of Emergency Medicine, New York, NY; ** Froedtert Hospital, Froedtert and the Medical College of Wisconsin Simulation Center, Milwaukee, WI

## Abstract

**Audience:**

This bleeding fistula model is designed to instruct emergency medicine residents and third- and fourth-year medical students on their emergency medicine rotation.

**Introduction:**

The prevalence of end-stage renal disease (ESRD) has increased since 2001 (808,536 people in 2021 versus 409,226 in 2001).^1^ About 14% of the United States population has a decreased glomerular filtration rate (GFR), and the overall presence of ESRD in the population as of 2021 was 2,219 per million population.^1^ As of 2021, 87.7% of patients receiving dialysis were on hemodialysis.^2^ Around 60% of dialysis patients use a fistula for access.^3^ Patients with ESRD have a high emergency department utilization rate, and emergencies related to dialysis include hyperkalemia, volume overload, bleeding at the dialysis site, infection, aneurysm, and pseudoaneurysm .^4^–^9^ It is important for emergency medicine (EM) physicians to be able to intervene quickly on life-threatening complications related to dialysis, including vascular access hemorrhage. There are approximately 250 deaths related to vascular access hemorrhages yearly, and it accounts for 0.4% of deaths in patients on dialysis.^4^ Patients with an initial presentation of hemorrhage from their vascular access site are also at risk for re-bleeding, and 80% of patients with bleeding die at home.^10^ Due to the high acuity and low occurrence (HALO) of bleeding fistulas, procedural knowledge of hemorrhage control and a thorough understanding of dialysis-related complications is paramount for EM physicians.

**Educational Objectives:**

After using the task trainer bleeding fistula model, learners will be able to: 1) identify vascular access hemorrhage as an emergency presentation in dialysis patients; 2) execute a stepwise approach to manage a bleeding fistula; 3) demonstrate effective hemorrhage control for a patient with uncontrolled bleeding from their fistula, including choice of appropriate suture material and suturing technique; and 4) discuss pitfalls of hemorrhage control in patients with fistulas, including risks of tourniquet use and complications related to clot formation at the fistula site.

**Educational Methods:**

The novel bleeding fistula model was embedded within a high-fidelity simulation for learners as part of weekly EM resident didactics. Learners received a pre-brief session to the simulation case. They then participated in a simulation scenario using a high-fidelity manikin with the bleeding fistula model on the manikin’s arm. The bleeding fistula model allowed learners to progress through a stepwise approach to achieve hemorrhage control in a patient presenting with bleeding from a dialysis access site. After the simulation, learners participated in a simulation debrief, which included a procedural skills workshop.

**Research Methods:**

This simulation scenario and model have been used in two academic year didactic sessions to collect learner data. Across the two years, a total of thirty-two learners consisting of fourth year medical students, post-graduate year 1 (PGY-1) and PGY-2 residents have participated in the high-fidelity simulation at a Society for Simulation in Healthcare (SSH) accredited, state-of-the-art simulation center. Learners performed a self-assessment survey using a three-point Likert scale after participating in the high-fidelity simulation case. A retrospective pre- and post-simulation survey was conducted. They answered survey questions related to their confidence of identification of a bleeding fistula and knowledge of treatment of a bleeding fistula before and after the simulation.

**Results:**

Over two years of implementation, we had thirty-two learners participate in the simulation and use the model for demonstration of hemorrhage control of a bleeding fistula. Based on self-assessment, learners overall felt that their overall knowledge regarding hemodialysis access hemorrhage increased. Prior to the simulation, eighteen learners rated their confidence in identification of a bleeding fistula as “average,” and fourteen learners rated their confidence as “below average.” After participating in the simulation, seventeen learners rated their confidence in identifying a bleeding fistula as “above average,” and fifteen learners rated their confidence as “average.” Prior to the simulation, thirteen learners rated their knowledge of the management of bleeding fistulas as “average,” and nineteen learners rated their knowledge of the management as “below average.” After the simulation, eighteen learners rated their knowledge in the management of a bleeding fistula as “above average,” and fourteen rated their knowledge of the management as “average.”

In the composite score, learners self-reported knowledge and confidence in managing bleeding fistulas improved. The pre-simulation score had a mean of 2.17 and a median of 2.0, increasing to a post-simulation mean of 3.31 and a median of 3.0. This difference was statistically significant (p < 0.00001), indicating a robust improvement in learners perceived comfort and knowledge following participation in the bleeding fistula simulation, hands-on task trainer, and debriefing workshop.

**Discussion:**

This novel fistula model helped residents practice a HALO procedure that closely simulated a real bleeding fistula. The model bled akin to a real fistula, with the ability to make the bleeding pulsatile and occlude with suturing, direct pressure, and tourniquet placement. In the literature review, there are no current simulation models or task trainers to get hands-on experience with management of hemodialysis access hemorrhage. This scenario tested resident knowledge of management of dialysis emergencies, and there was a good discussion regarding the subject. Residents stated that this simulation was helpful. They learned new information and were able to get hands-on practice to reinforce that knowledge through this activity and the use of the bleeding fistula model.

**Topics:**

Dialysis, end stage renal disease, end stage renal disease complications, bleeding fistula, hemorrhage control, simulation, fistula model, HALO Procedure.

## USER GUIDE

**Table:** 

List of Resources:	
Abstract	1
User Guide	4
Appendix A: Model Materials, Cost, and Suggested Alternatives	11
Appendix B: Learner Pearls/Case Takeaways	14
Appendix C: Discussion Notes for Instructor	15
Appendix D: PowerPoint Presentation	20

**Learner Audience:**

This simulation is appropriate for medical students, interns, and junior residents.

**Time Required for Implementation:**

Creating the task trainer model is quick and easy. Once the plan for the model was developed, the simulation team took under an hour to create the model and set up the model on a manikin. This included time to build four individual fistula models, the simulation fistula, and three task trainer fistula models, as required for the number of learners present. We estimate that it would take approximately 30 minutes to build a single model.

Learners can become proficient in management of dialysis-related hemorrhage with this model in approximately 30 minutes. If used as a task trainer, each learner would need about 30 minutes to practice their skills. If embedded in a simulation, we would recommend having a total of 90 minutes for the case. This would include about 30 minutes for the simulation, 30 minutes for the debrief, and 30 minutes for the hands-on skills practice to develop procedural knowledge and skills.

**Recommended Number of Learners per Instructor:**

One learner at a time can use the model. With repetitive use to practice, the model would maintain its fidelity for five individual learners. The skin would then have to be exchanged. One instructor is sufficient to teach learners about fistula hemorrhage with the model. To run a simulation, it is helpful to have additional simulation-trained instructors or content experts to facilitate the pre-brief and debrief. Ideally, one or two additional instructors for a high-fidelity simulation would be appropriate.

**Topics:**

Dialysis, end stage renal disease, end stage renal disease complications, bleeding fistula, hemorrhage control, simulation, fistula model, HALO Procedure.

**Objectives:**

After using the task trainer bleeding fistula model, learners will be able to:

1. Identify vascular access hemorrhage as an emergency presentation in dialysis patients.
2. Execute a stepwise approach to manage a bleeding fistula.
3. Demonstrate effective hemorrhage control for a patient with uncontrolled bleeding from their fistula, including choice of appropriate suture material and suturing technique.
4. Discuss pitfalls of hemorrhage control in patients with fistulas, including risks of tourniquet use and complications related to clot formation at the fistula site.

### Linked objectives, methods and results

Using the Learner PEARLS/Case Takeaways (Appendix B), Discussion Notes for Instructors (Appendix C), and PowerPoint Presentation (Appendix D), along with the fistula model, the learning objectives can be met through an interactive didactic lecture and hands-on task trainer demonstration. The didactic lecture can occur either before or after the hands-on task trainer demonstration. Learners will review emergency presentations of dialysis patients, including vascular access hemorrhage. They then identify a stepwise approach to management of a bleeding fistula and discuss pitfalls of hemorrhage control. Finally, using the fistula model, learners will demonstrate effective hemorrhage control.

Alternatively, the fistula model could be embedded into a simulation case. This method can create a high-fidelity and psychologically safe environment to have learners execute a stepwise approach and demonstrate effective hemorrhage control. The simulation debrief would address the learning objectives through facilitator led discussion of dialysis access hemorrhage emergencies, stepwise approach to management, discussion of hemorrhage control options, and pitfalls of hemorrhage control.

### Recommended pre-reading for instructor

- Bleeding Dialysis Fistula | CorePendium^4^
- End-Stage Renal Disease | Tintinalli's Emergency Medicine: A Comprehensive Study Guide, 9^th^ ed^5^
- Bleeding AV Access^6^
- EM DOCs | Dialysis The Dialysis Patient: Managing Fistula Complications in the Emergency Department^7^
- Renal Failure | Rosen’s Emergency Medicine: Concepts and Clinical Practice, 10^th^ ed^9^

### Learner responsible content (LRC)

There were no assignments prior to the case to maintain the fidelity of the simulation case. This list of resources was provided to the learners afterwards. The pre-reading above for the instructor could also be provided to the learners beforehand. Please see Appendix B and C for detailed learner takeaways and discussion notes for instructors.

### Additional educational materials

- EMRAP Podcast: Suturing a Bleeding Dialysis Fistula^11^
- EMRAP Podcast: Bleeding AV Shunt^12^

### Implementation Methods

This model can be used alone as a task trainer or embedded as a part of a simulation case. A case was developed around dialysis hemorrhage, and the model was developed in conjunction with that case, which was run in EM didactics in a simulation center using a high-fidelity simulation mannequin. The model was applied directly to the mannequin. Active learners participated in the simulation; they assigned tasks within the simulation prior to participation. During the simulation, additional learners observed using a one-way mirror and video recording. Following the simulation, all learners participated in the simulation debrief to discuss management of dialysis access hemorrhage. Following the debrief, participants were given the opportunity to practice their skills with the task trainer.

Alternatively, the fistula model could be used with a standardized patient or as a stand-alone task trainer. In a standardized patient, the same simulation case would be recommended. As a task trainer, the model could be used with or without an IV arm. A brief presentation reviewing the management of a bleeding fistula and appropriate suture techniques could occur before or after task trainer use, based on learner level and experience to address the learning objectives.

### List of items required to replicate this innovation

**Table:** 

Item Used	Cost and Vendor
Simulated blood	$180/2 gallon container from Strategic Operations
Silicone arm sleeve	$316 from Strategic Operations (IV and suture sleeve)
Suture pad for fistula	$75/each from SIMUlab (each suture pad makes about 6 fistulas)
Vein tubing ⅛” internal diameter	$18/104 inch from Gaumard (veins consumable for venous training arm)
Female Luer lock adapter	$8.99/10 pieces from Amazon MEETOOT 10 pcs Female Luer Lock 1/8″ Polycarbonate Hose Barb Adapter
Tubing clamp	$11.99 for 20 purchased from Amazon
500 ml fluid bag	$1.88 each ordered through hospital distribution (0.9% NaCL 500ml bag)
Secondary tubing to spike fluid bag	$1.17 each ordered through hospital distribution (secondary Alaris Pump tubing)

### Approximate cost of items to create this innovation

The total approximate cost for this model built from scratch and using high-fidelity simulation equipment is $615.00. This total cost includes all materials necessary to build a single model. Apart from the silicon arm sleeve, the purchased equipment used to build the model will have extra material left over that can be used for a second fistula model or other simulations. Once completely built, the model can be used multiple times. It is recommended that the suture pad be exchanged after five learners use the model. The suture pad that was purchased for this model is enough to exchange it six times, allowing for a total of thirty learners to use the model. This would bring the cost of the material for the construction to approximately $21 per learner. Suture materials, tourniquet, and bandage equipment were provided separately and not included in the cost of building the model. Some products were purchased specifically for the development of this model, while others were part of the stock in the simulation center in which the model was developed. Many of the items came as part of a set purchased from Strategic Operations, a company that sells training and moulage for simulation performed by military, law enforcement, and healthcare. Other supplies were purchased through other vendors, including Amazon and the facility’s company distribution department. It would be possible to cut the cost of the model significantly by using alternative equipment. A detailed list of specific materials, costs, and alternatives is outlined in Appendix A.

### Detailed methods to construct this innovation

**Image 1:**
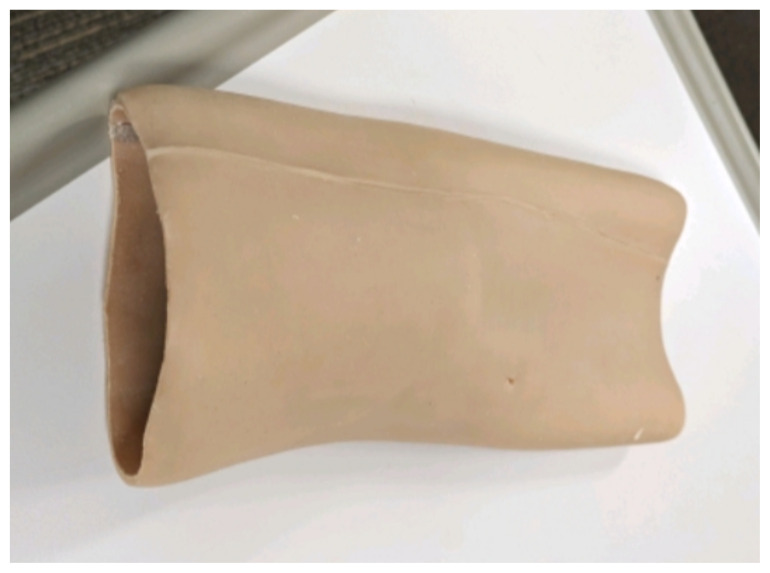
Silicone arm sleeve: Author’s own image.

**Image 2:**
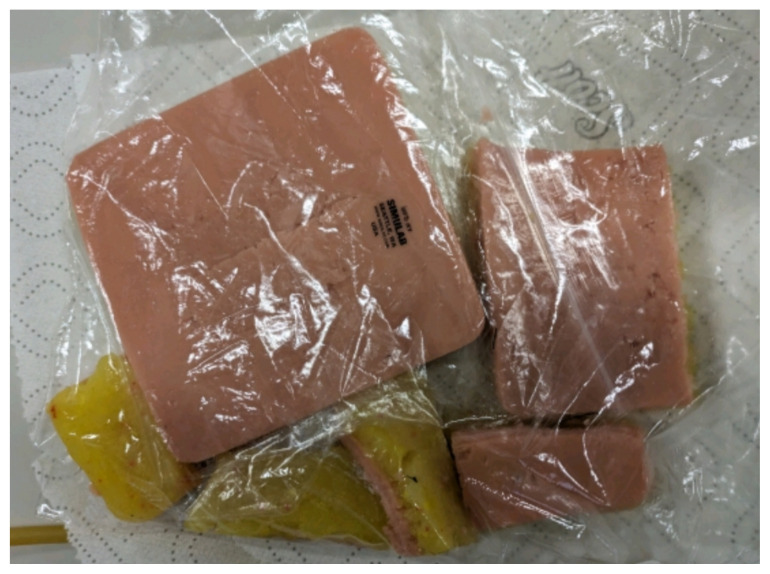
Suture pad: Author’s own image.

**Image 3:**
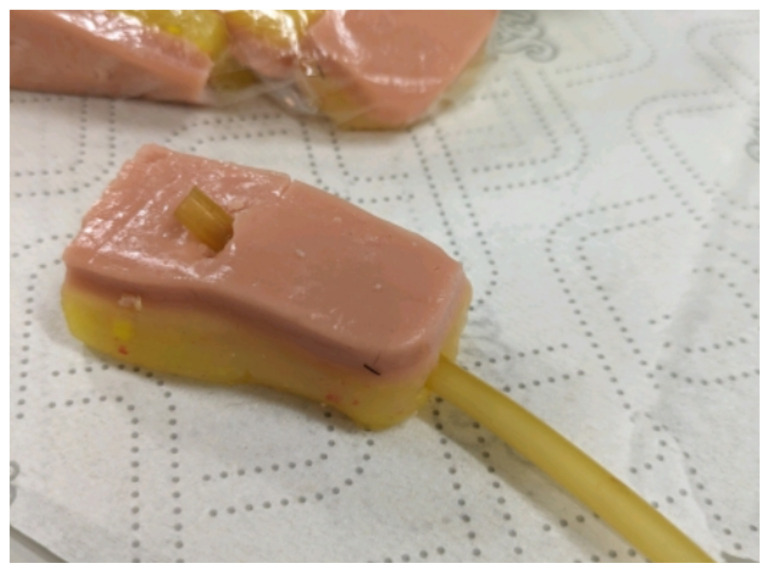
Tubing in suture pad: Author’s own image.

**Image 4:**
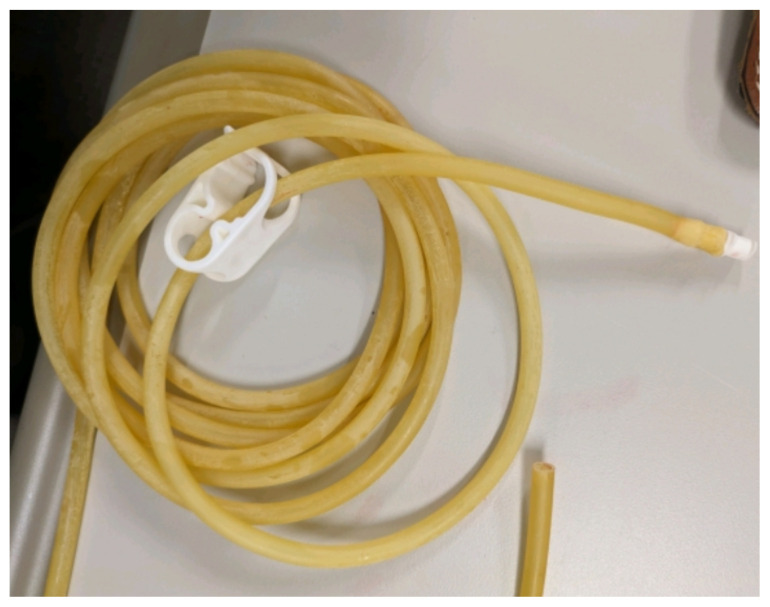
Blood tubing: Author’s own image.

**Image 5:**
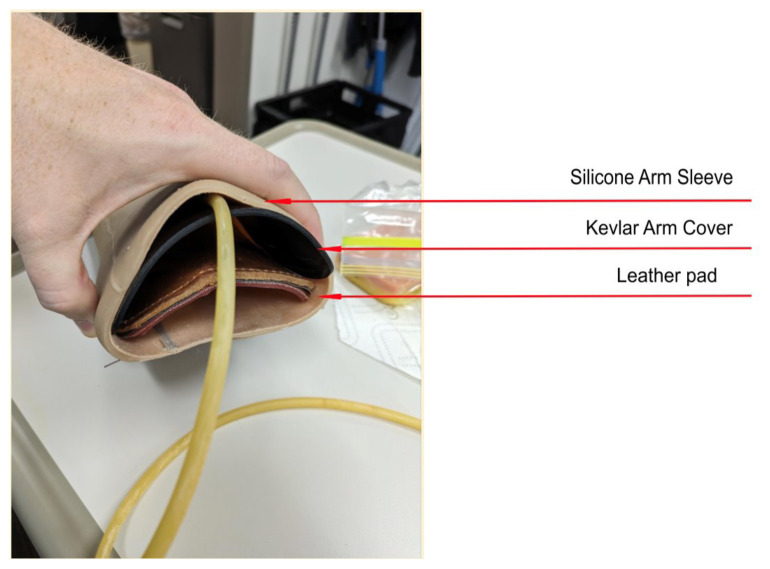
Side view of fistula model with leather sheet and Kevlar arm cover: Author’s own image

**Image 6:**
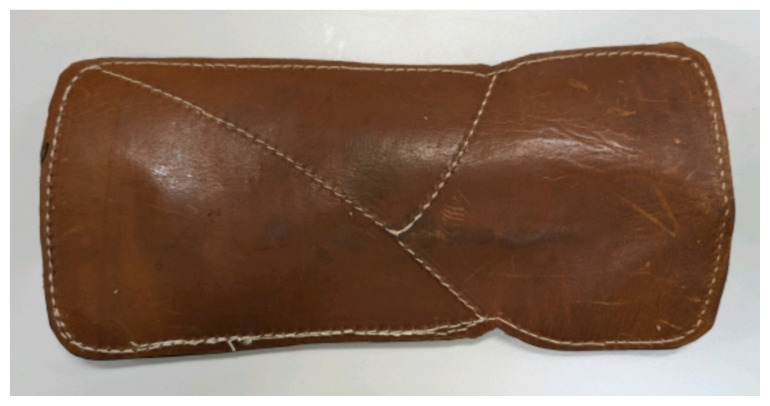
Leather sheet: Author’s own image.

**Image 7:**
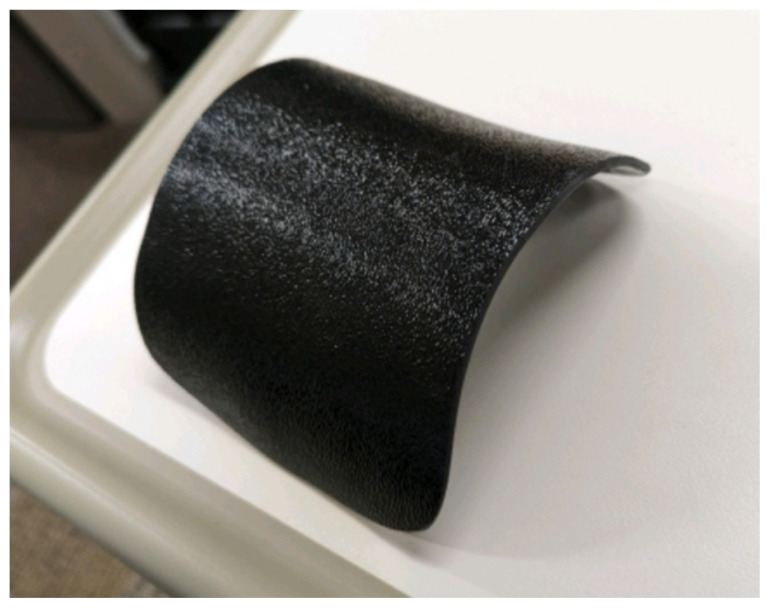
Kevlar arm cover: Author’s own image.

**Image 8:**
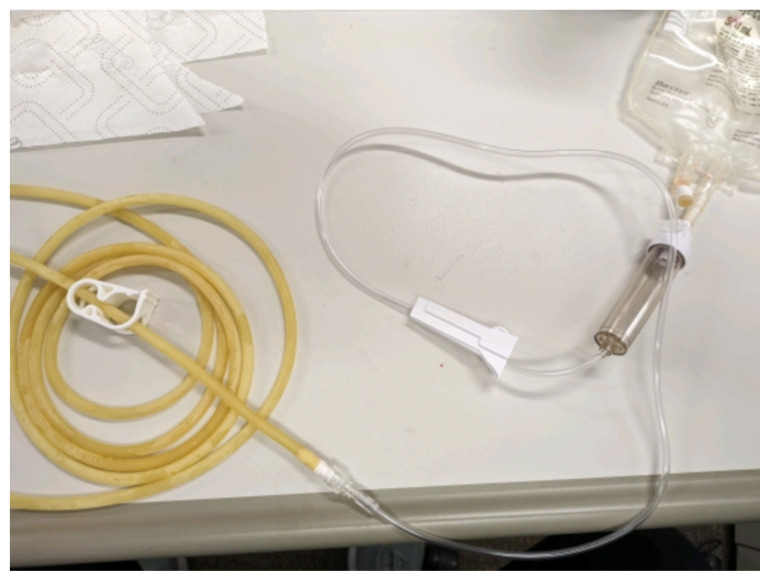
Connection to blood source: Author’s own image.

**Image 9:**
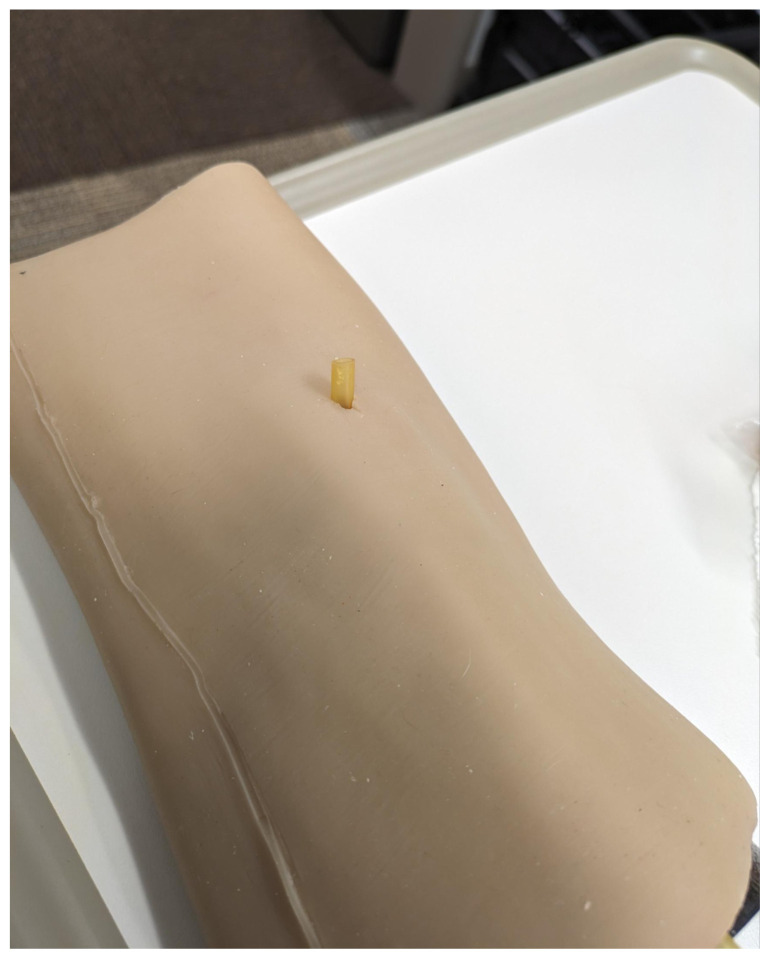
Final fistula model: Author’s own image.

**Image 10:**
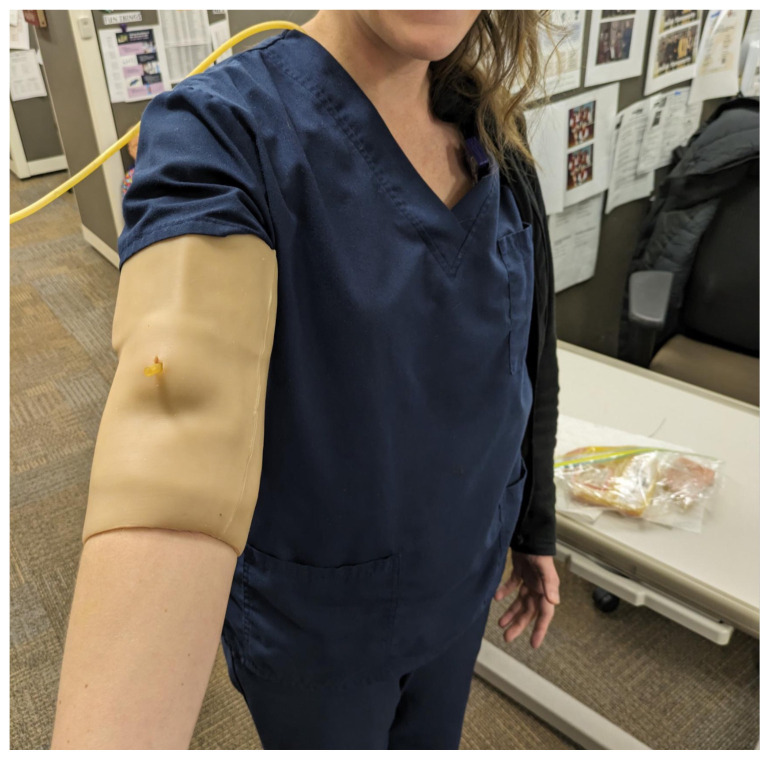
Fistula model on standardized patient: Author’s own image.

**Image 11:**
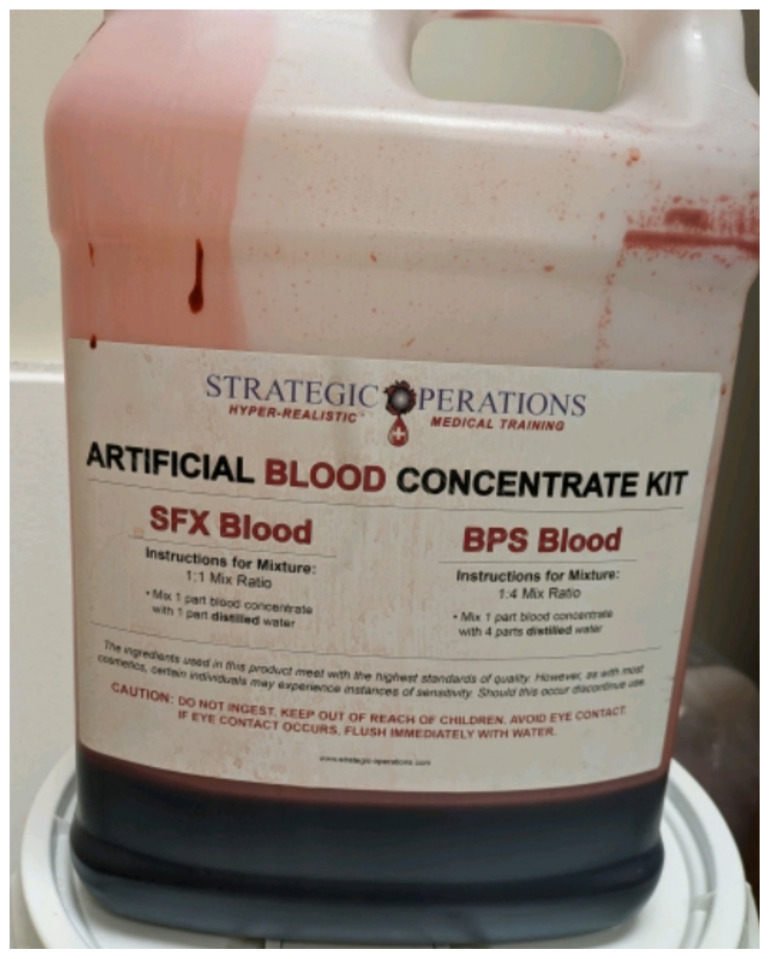
Strategic Operations Artificial Blood Concentrate Kit: Author’s own image.

1. Obtain a premade silicone arm sleeve that comes with a full kit from Strategic Operations (Image 1).
  *Best practice and alternative considerations:* The sleeve was purchased from Strategic Operations. The silicone sleeve has a similar feel to skin, stretches, is easy to clean, and can be reused if providing appropriate care. Premade simulation items can be expensive, so a neoprene arm sleeve or brace that is close to the skin tone could be considered. It is important to size up on the sleeve or brace to allow room for the fistula underneath.
2. Cut a small segment from a suture pad for the fistula, roughly 2½ inches x 1 inch (Image 2).
  *Best practice and alternative considerations:* The suture pad is from Simulab and has a skin layer and fatty tissue layer. There are several other items that could be used in lieu of a Simulab suture pad including suture pad trainers available through Amazon or dense memory foam, if there are budgetary restrictions (see Appendix A).
3. Using a small Phillips-head screwdriver or other sharp instrument, create a hole in the suture pad to run the tubing through to simulate the vessel. The initiation of the path starts on the proximal side of fistula, directly below the epidermis. Halfway through the suture pad segment, the course changes, so that the hole exits the surface of the skin where the bleeding will occur (Image 3).
4. Thread the tubing through the track created in the suture pad (Image 3).
  *Best practice and alternative considerations:* The tubing used for the vessel is replacement line tubing for the Gaumard phlebotomy arm (Image 4). Multiple lines and tubing were tested, and this was the best option for multiple reasons. First, the tubing needs to have a large enough bore size to allow appropriate blood flow but cannot be so large it creates a large hole in the arm sleeve and suture pad. We recommend using tubing with an internal diameter of 1/8” and external diameter of 3/16.” The tubing also cannot be too rigid; it must be compressible enough to fully occlude when a tourniquet or manual pressure is applied. Finally, the tubing needs to have sufficient length. It should be long enough to run up the standardized patient or manikin’s arm, through a gown, and then to a blood source with enough slack to hang high enough for gravity to assist in blood flow.
5. Layer the components (arm sleeve, suture pad with venous tubing, and arm barrier) of the task trainer together (Image 5).
  *Best practice and alternative considerations:* To protect either the standardized patient or manikin, we used a thick leather sheet as protection from suturing (Image 6). A polymer/Kevlar arm cover that came with a kit from Strategic Operations was also used in this case for protection from the potential use of a tourniquet (Image 7). Be mindful to have the vessel run over the top of the arm protector so that it is between the arm protection and the skin on the sleeve. This way it will fully occlude with appropriate compression.
6. Finally, connect the fistula trainer to the blood source (Image 8). To connect the trainer to the blood source, a female Luer lock adapter with a barb at the end of the vessel tubing was used. The secondary Alaris pump tubing then connected to the vessel and was spiked into the blood bag. Since the tubing has a rolling clamp, the flow rate can be set. A second tubing clamp can be used to clamp off the flow until ready. Once completed, the final model is a reusable, suturable, bleeding fistula which can be worn by a live standardized patient, added to a manikin, or used as a stand-alone task trainer (Image 9, Image 10).
  *Best practice and alternative considerations:* The bleeding could be made to be pulsatile by squeezing the blood bag, or by using a blood filled 50–60ml Luer lock syringe instead of the secondary tubing and blood bag.
  The blood that we used is Strategic Operations Artificial Blood Concentrate Kit (Image 11). It is deep in color, has great viscosity, does not stain, and is washable. It also cleans out easily from tubing, using vinegar and distilled water. When creating the blood for purposes of running through a tube, use the Blood Pumping System (BPS) Blood instructions for mixture, found on the container.

### Results and tips for successful implementation

The fistula model was embedded in a simulation case of a patient presenting after hemodialysis with bleeding from the fistula site where learners needed to demonstrate hemorrhage control. A post-session survey was used to gauge learner confidence and knowledge with dialysis complications and success of the bleeding fistula model. There was not a formal competency test associated with the session.

Over two years of implementation, we had a total of thirty-two learners participate in the simulation and use the model for demonstration of hemorrhage control of a bleeding fistula. Based on the self-assessment, learners overall felt that their overall knowledge regarding hemodialysis access hemorrhage increased. Prior to the simulation, eighteen learners rated their confidence in identification of a bleeding fistula as “average,” and fourteen learners rated their confidence as “below average.” After participating in the simulation, seventeen learners rated their confidence in identification of a bleeding fistula as “above average,” and fifteen learners rated their confidence as “average.” Prior to the simulation, thirteen learners rated their knowledge of the management of bleeding fistulas as “average,” and nineteen learners rated their knowledge of the management as “below average.” After the simulation, eighteen learners rated their knowledge in the management of a bleeding fistula as “above average,” and fourteen rated their knowledge of the management as “average.”

In the composite score, learners self-reported knowledge and confidence in managing bleeding fistulas improved. The pre-simulation score had a mean of 2.17 and a median of 2.0, increasing to a post-simulation mean of 3.31 and a median of 3.0. This difference was statistically significant (p < 0.00001), indicating a robust improvement in learners’ perceived comfort and knowledge following participation in the bleeding fistula simulation, hands-on task trainer, and debriefing workshop.

Generally, this education was well received. However, our team identified a few improvements for future directions. There was a time constraint due to other simulation sessions occurring at the same time. Sixty minutes were provided for the simulation, debrief, and task trainer practice. It would have been helpful to have an additional thirty minutes for further hands-on practice. Additionally, the participants in this group were fourth-year medical students, PGY-1 and PGY-2 emergency medicine learners. This case was appropriately leveled and may be too simple for a more advanced learner.

Though this model could be used as a stand-alone task trainer, it is recommended as an embedded portion within a simulation case with either a manikin or standardized patient. Simulation creates a more realistic scenario, where learners can see the patient’s vital signs change based on their clinical progression and interventions performed. It more closely replicates an encounter in the emergency department when compared with using a task trainer alone. Though it can be more stressful for learners, it is a psychologically safe environment for learners to test their knowledge and demonstrate procedural techniques in a high-fidelity setting. Simulation also allows learners to practice important skills related to managing critically ill patients including closed loop communication, teamwork, and leadership.

### Associated content

- Appendix A: Model Materials, Cost, and Suggested Alternatives
- Appendix B: Learner Pearls/Case Takeaways
- Appendix C: Discussion Notes for Instructor
- Appendix D: PowerPoint Presentation

## Supplementary Information



## Figures and Tables

**Figure:**
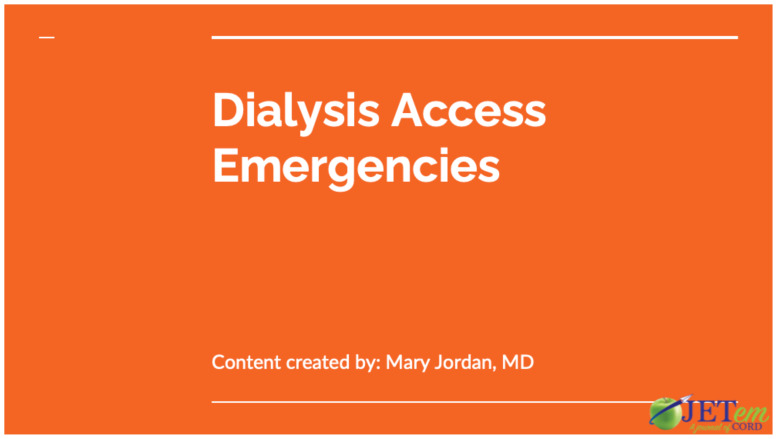
Please see associated PowerPoint file

## INNOVATION MATERIALS

### Appendix A Model Materials, Cost, and Suggested Alternatives

The following are costs for items we used while making this trainer as well as suggested alternatives to certain items for cost savings.

**Table:**

Item Used	Cost and Vendor	Usage Notes and Advantages	Suggested Alternative	Usage Notes
Simulated blood	$180/2 gallon from Strategic Operations.	Only a few ounces are used and will last a week or two. Does not stain material.	Water and red food coloring.	Not as thick as simulated blood. Will stain materials.
Silicone arm sleeve	$316 from Strategic Operations (IV and suture sleeve).	Easy to repair with silicone for many uses. Flexible and feels like skin.	Neoprene arm sleeve, compression band, or Ace wrap.	Not as easily repaired as silicone sleeve and not as life-like.
Plastic/polymer arm guard	Included with the arm sleeve from Strategic Operations.	Meant to protect a standardized patient from accidental needle poke during suturing. Also to protect a standardized patient from arterial occlusion during tourniquet application.	Anything that forms to the manikin or standardized patient’s arm that will not break or cause damage to the wearer if a tourniquet is applied.	
Suture pad for fistula	$75/each from SIMUlab (each suture pad makes about 6 fistulas).	The suture pads provide multiple layers for suturing and it is very easy to run the vein tubing through.	Silly putty or memory foam.	Material needs to be thick enough to run a suture needle through and have the tubing housed in it.
Vein tubing	$18/104 in. from Gaumard (veins consumable for venous training arm).		With all the tubing tested in building this model, this was the best for what is needed and is comparable in price; therefore, we have no suggested alternatives.	
Female Luer lock adapter	10 pcs/$8.99 From Amazon MEETOOT 10 pcs Female Luer Lock 1/8″ Polycarbonate Hose Barb Adapter.		No suggested alternatives.	
Tubing clamp	$11.99 for 20 purchased from Amazon.		No suggested alternatives.	
500ml fluid bag	$1.88 each ordered through our hospital distribution (0.9 NaCL 500ml bag).			
Secondary tubing to spike fluid bag	$1.17 each ordered through our hospital distribution (secondary Alaris Pump tubing).			

### Appendix B Learner PEARLS/Case Takeaways

1. Types of dialysis related emergencies:
  - Hyperkalemia
  - Volume overload
  - Bleeding access site
    - Approximately 250 deaths/year
  - Infection
  - Aneurysm
  - Pseudoaneurysm
2. Hemorrhage control approach
  - Direct pressure
  - Topical hemostatic medications
  - Suture
  - If bleeding successfully controlled, observe for 1–2 hours
  - Last resort: tourniquet
    - Complications related to tourniquet: fistula thrombosis
3. What requires a consult
  - Aneurysm, pseudoaneurysm
  - Signs of infection
    - Cover broadly with MRSA and gram-negative coverage
  - Ulceration
  - Thinned skin
  - Only hemorrhage control is a tourniquet
  - Recurrent bleeding
  - Loss of a palpable thrill at fistula site

### Appendix C Discussion Notes for Instructor

1. Identify specific emergencies related to dialysis
  - Dialysis access types:
    - Fistula- connection between artery and vein without exogenous material
      1. AV Fistula^13^
    - Graft--donor vein to create connection or synthetic
  - Types of emergencies: hyperkalemia, volume overload, bleeding fistula/graft, infection, aneurysm, pseudoaneurysm
    - Aneurysm - dilation of all 3 layers of vessel wall
    - Pseudoaneurysm - extramural pulsatile hematoma with blood flow walled off in the soft tissue
  - Bleeding emergency: increased risks
    - Approximately 250 deaths per year
    - Grafts have a greater risk than fistulas for complications including bleeding, ~ 6x relative risk
    - Worse with:
      1. Coagulopathy
      2. Increased intraluminal pressure from proximal vein stenosis (creates outflow obstruction, causes increased intraluminal pressure, increases risk of bleeding)
      3. Infection at the site
      4. Hypertension
      5. Repeat trauma
2. Stepwise Approach for Bleeding Control
  - Direct Pressure
    - Establish IV access, in case treatment for hemorrhagic shock is needed
    - Apply pressure with 1 finger or a fistula clamp (broad pressure/bulky dressing is too diffuse and allows for continued bleeding)
      1. Additionally want to maintain the underlying thrill so as not to cause thrombosis
    - Apply pressure for 10–20 min
    - If hemostasis achieved, check for palpable thrill; if no longer present consult a vascular surgeon
  - Topical hemostatic agents: gelatin sponges, microfibrillar collagen, oxidized regenerated cellulose, tranexamic acid solution–soaked gauze, topical thrombin either on gauze or on prothrombotic matrix, and chitosan
    - Gelfoam--water insoluble sponge from porcine skin, gelatin granules and water, creates a mechanical matrix
      1. Apply bandage once hemostasis is achieved
    - Chitosan--complex carbohydrate derived from Chitin
    - Thrombins
      1. Activates XI, VIII, V, XII and I to facilitate hemostasis
  - Suture technique
    - Indication: Uncontrolled bleeding from a hemodialysis AV fistula/graft
    - Suture type: 4-0 monofilament, ie, nylon, polypropylene
    - Needle type: taper, NO cutting needles
      1. Taper needle--round, designed to separate fibers rather than cut
      2. Cutting--cutting edge on inside of circle
      3. Reverse cutting--cutting edge on outside of circle
      4. Needle Types: Author’s own image
    - Suture type:
      1. Figure 8 suture
        - Figure 8 Suture: Author’s own image
      2. Horizontal mattress or purse string suture
        - Purse String Suture^14^
      3. Vascular closure button
        - Vascular Closure Device^15^
    - Goal: Tie only as much as you need to be effective without causing skin necrosis
  - Tourniquet--avoid unless bleeding is truly uncontrolled because it can lead to complications including thrombosis and possible need for new dialysis access
  - If successfully able to control bleeding, observe for 1–2 hours afterward
3. Additional considerations for bleeding (ie, coagulopathy)
  - Anticoagulant reversal
    - Prothrombin complex concentrate (PCC) for Factor Xa inhibitors
    - Vitamin K and PCC for warfarin
    - Idarucizumab for dabigatran
  - Desmopressin (DDAVP) 0.3 ug/kg for bleeding in the setting of uremia
  - Protamine--consider use of protamine if heparin is used during dialysis: 1 mg for every 100 mg heparin, 10–20 mg total
4. What requires a consult
  - Aneurysm, pseudoaneurysm
  - Signs of infection--cover broadly with MRSA and gram-negative coverage
  - Ulceration
  - Thinned skin
  - Only hemorrhage control is a tourniquet
  - Recurrent bleeding
  - Loss of a palpable thrill at fistula site after pressure is applied
