# Supplementary material for: Stopping Fistula Hemorrhage without Bleeding Time and Money - A Low Cost, Low Resource Hemodialysis Fistula Model for Emergency Medicine Residents
Source: J Educ Teach Emerg Med. 2026 Apr 30;11(2):I1–I20. doi: 10.5070/M5.52204 (PMC13152352; doi:10.5070/M5.52204)
Supplement: Supplementary file 1 [file 11-2-I1-Appendix_D.pptx]

## Slide 1
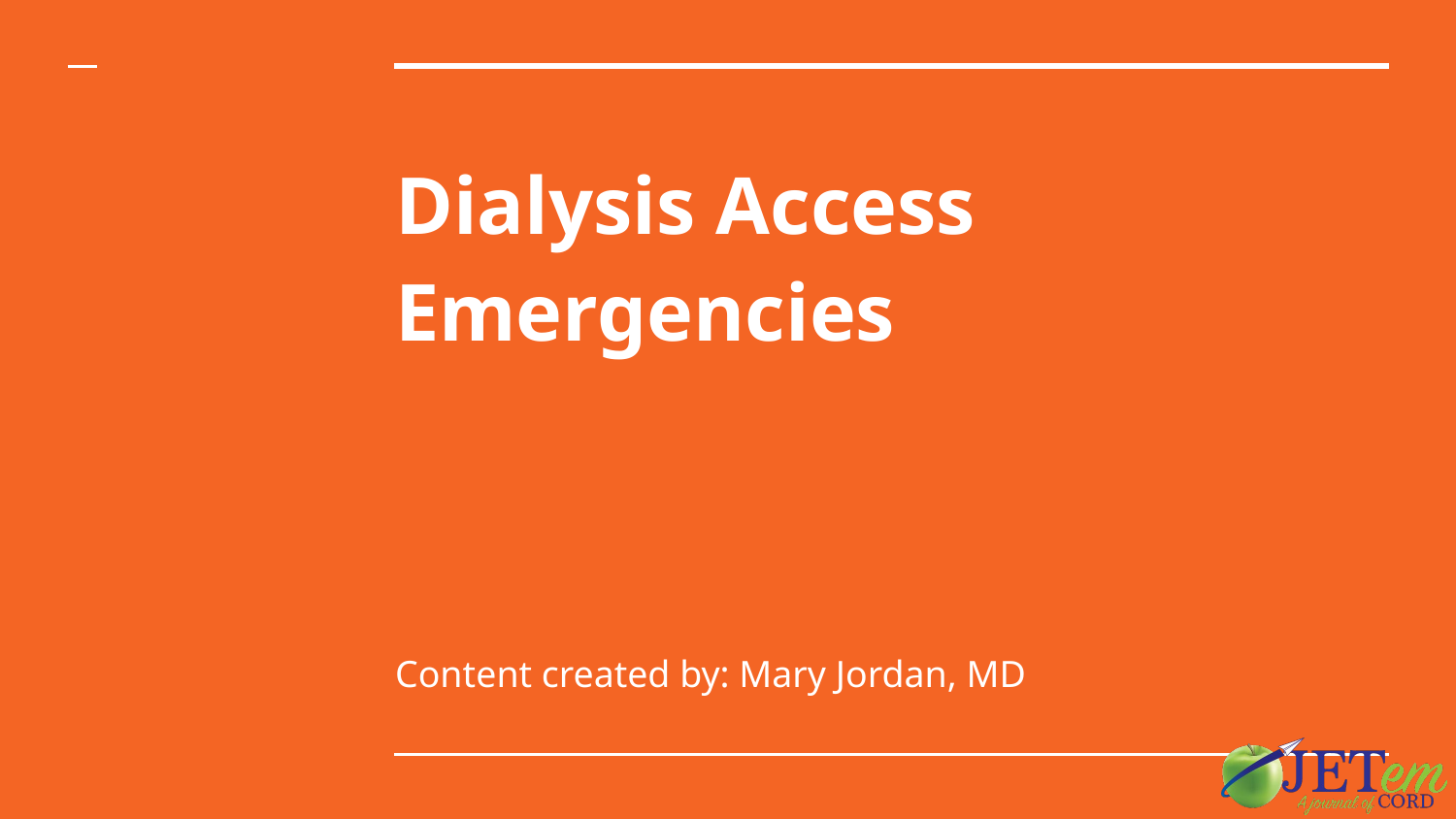

# Dialysis Access Emergencies
Content created by: Mary Jordan, MD

## Slide 2
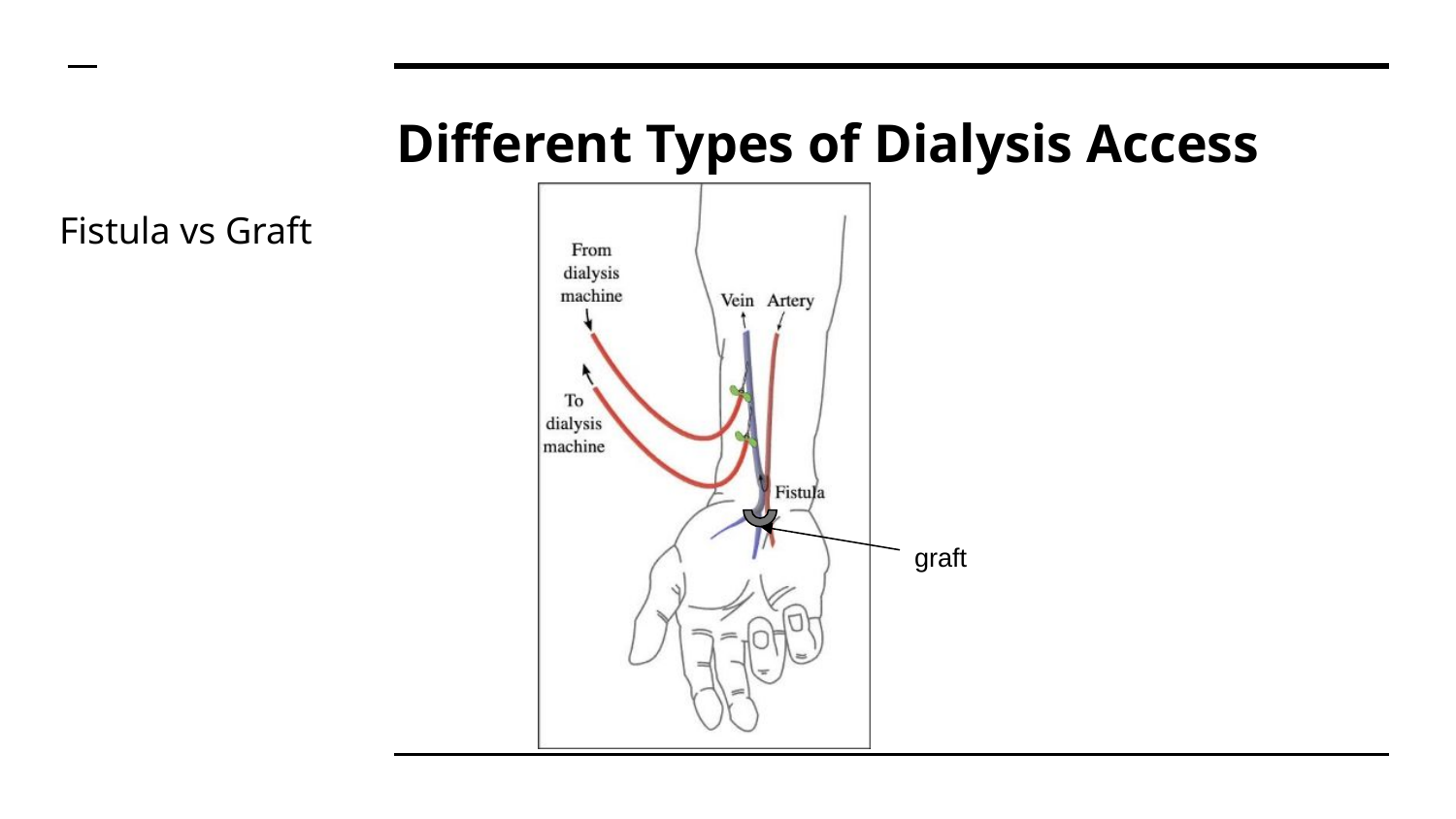

# Different Types of Dialysis Access
Fistula vs Graft
graft

## Slide 3
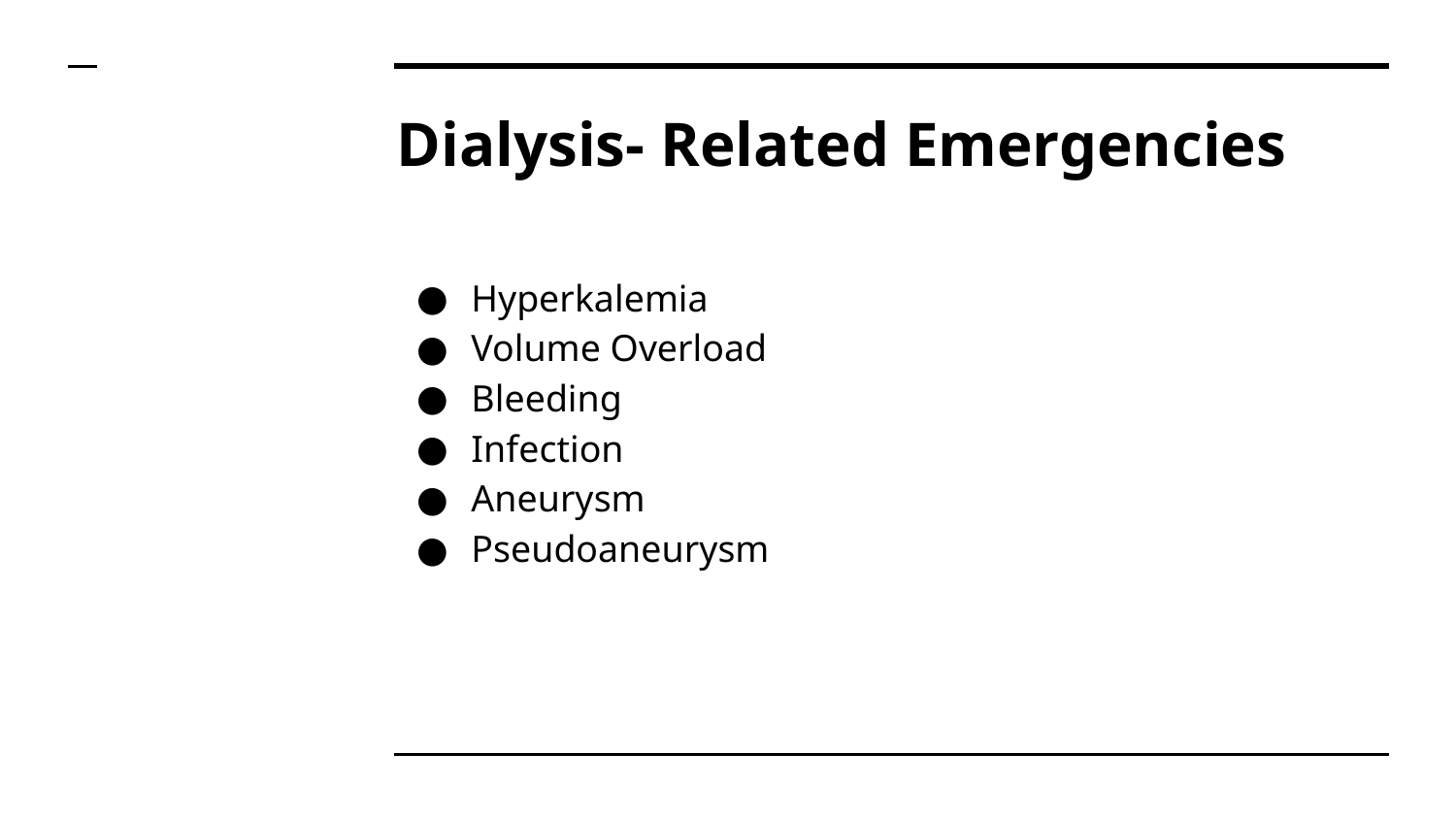

# Dialysis- Related Emergencies
Hyperkalemia
Volume Overload
Bleeding
Infection
Aneurysm
Pseudoaneurysm

## Slide 4
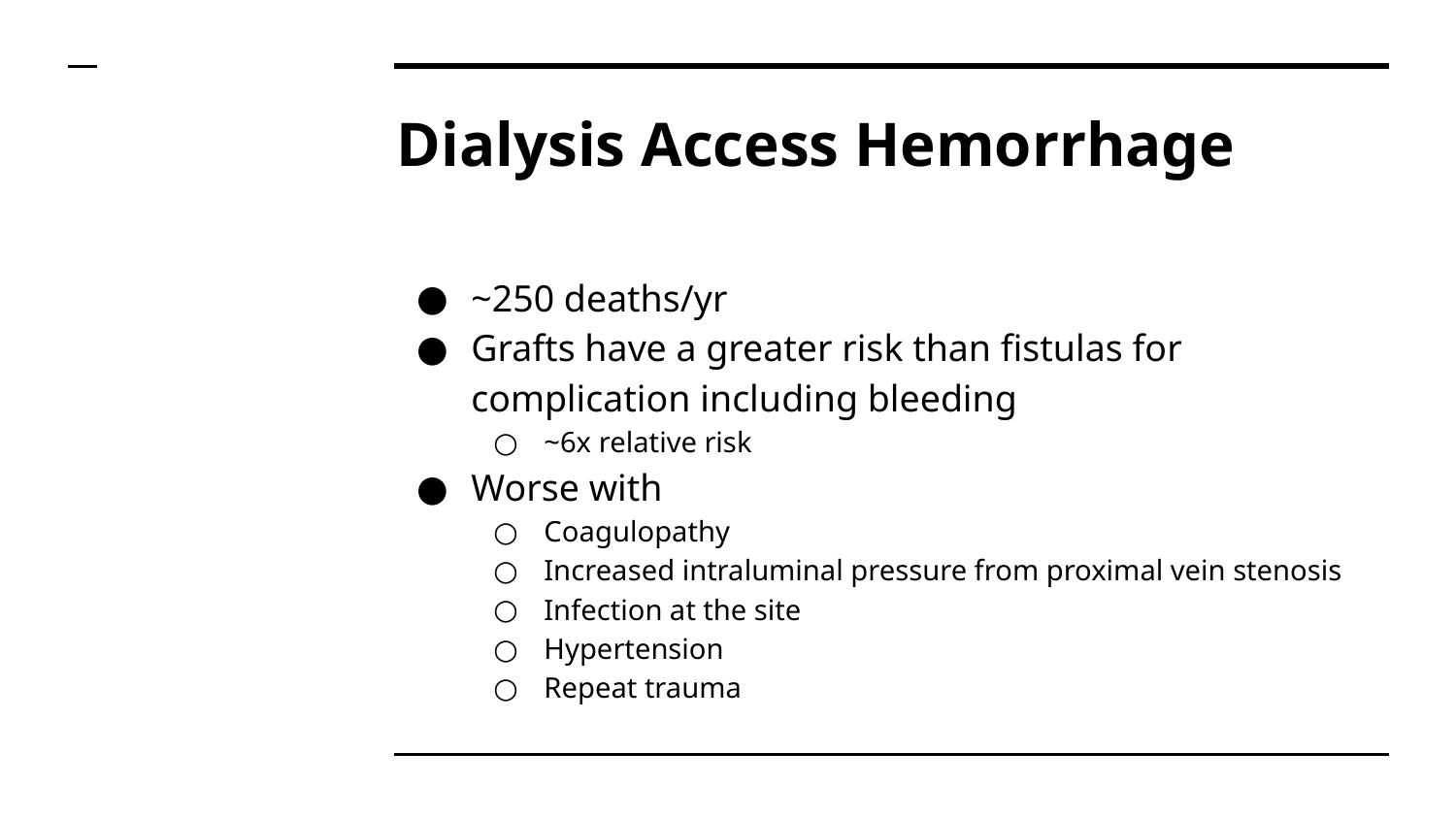

# Dialysis Access Hemorrhage
~250 deaths/yr
Grafts have a greater risk than fistulas for complication including bleeding
~6x relative risk
Worse with
Coagulopathy
Increased intraluminal pressure from proximal vein stenosis
Infection at the site
Hypertension
Repeat trauma

## Slide 5
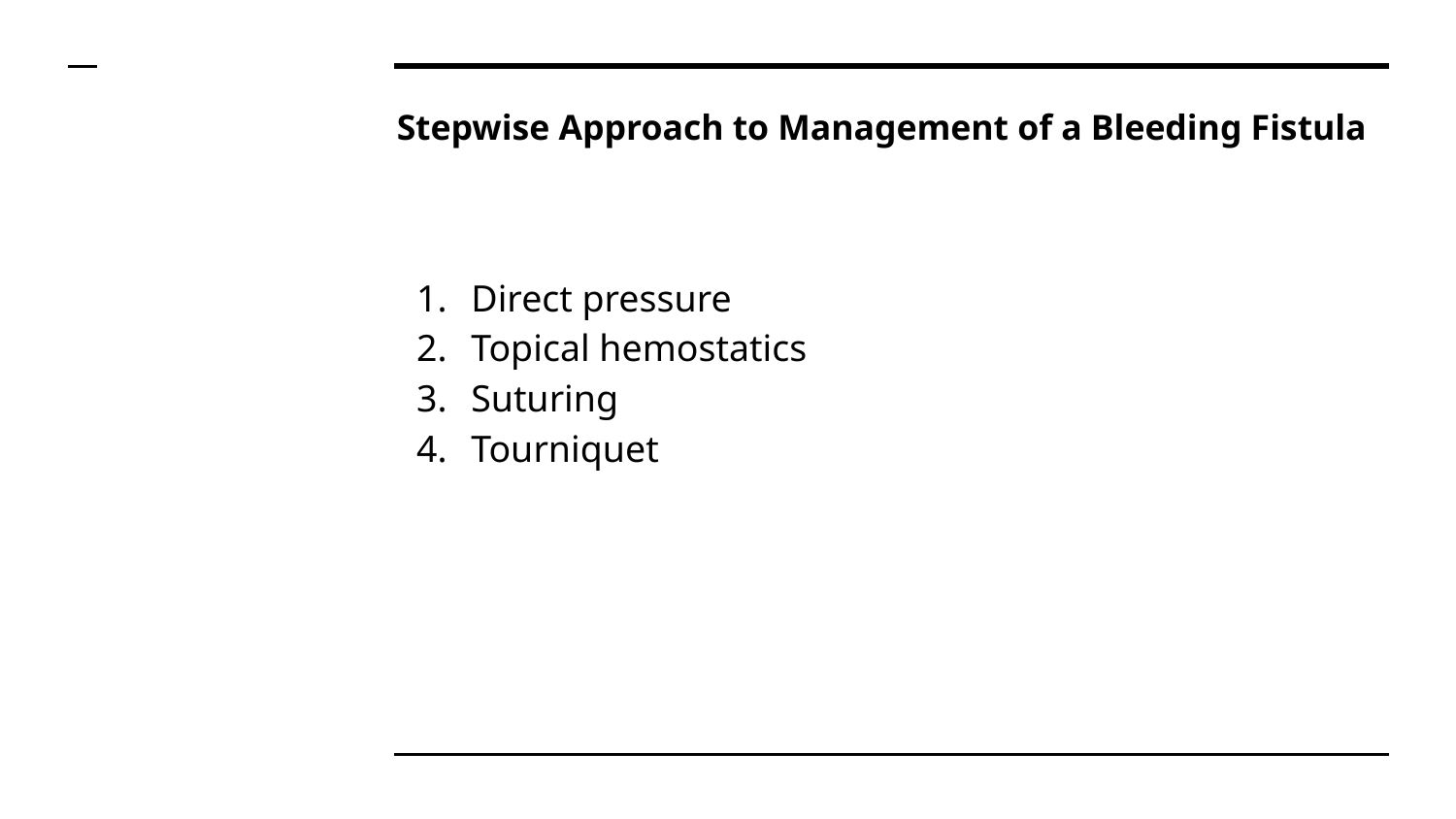

# Stepwise Approach to Management of a Bleeding Fistula
Direct pressure
Topical hemostatics
Suturing
Tourniquet

## Slide 6
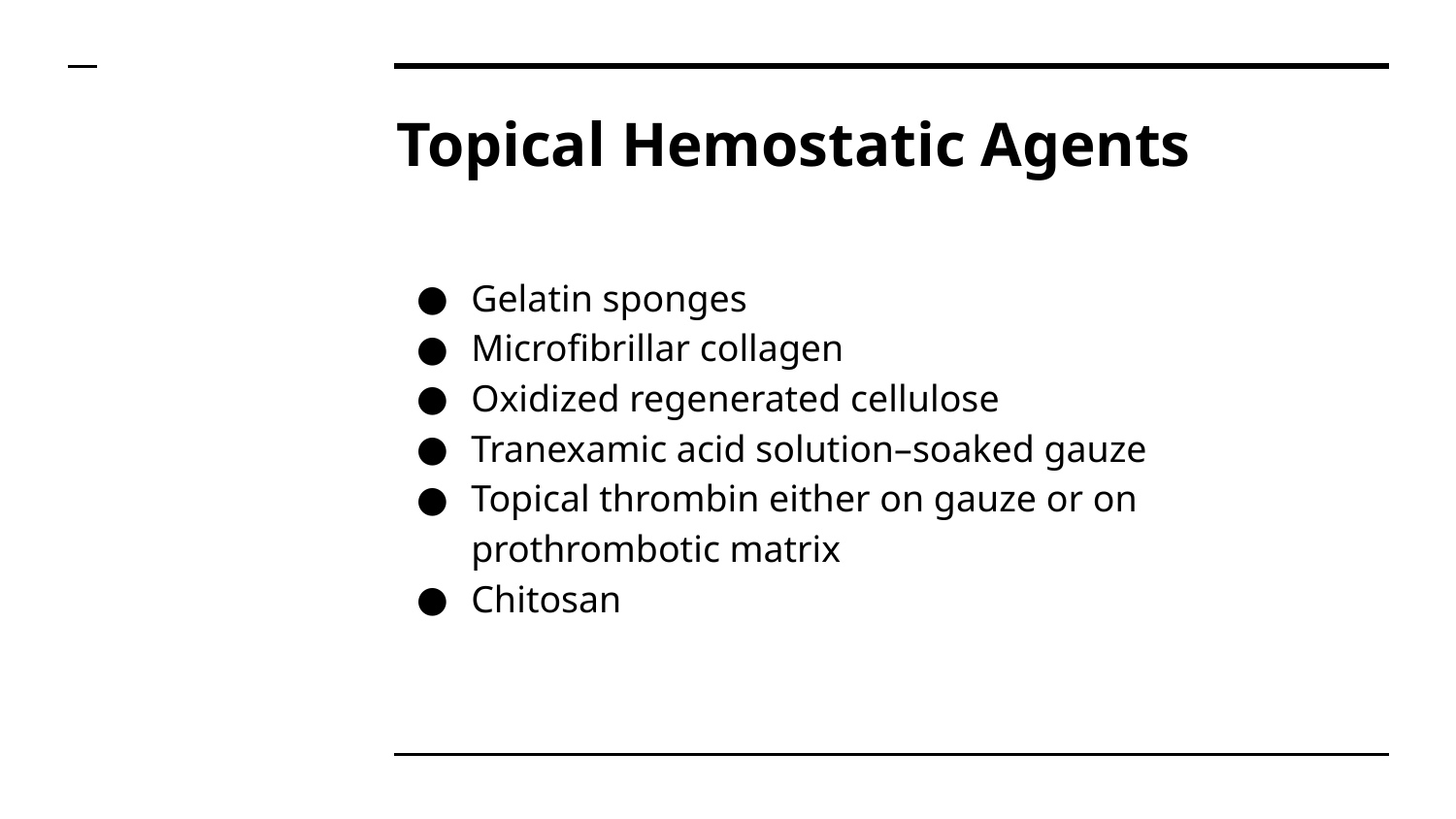

# Topical Hemostatic Agents
Gelatin sponges
Microfibrillar collagen
Oxidized regenerated cellulose
Tranexamic acid solution–soaked gauze
Topical thrombin either on gauze or on prothrombotic matrix
Chitosan

## Slide 7
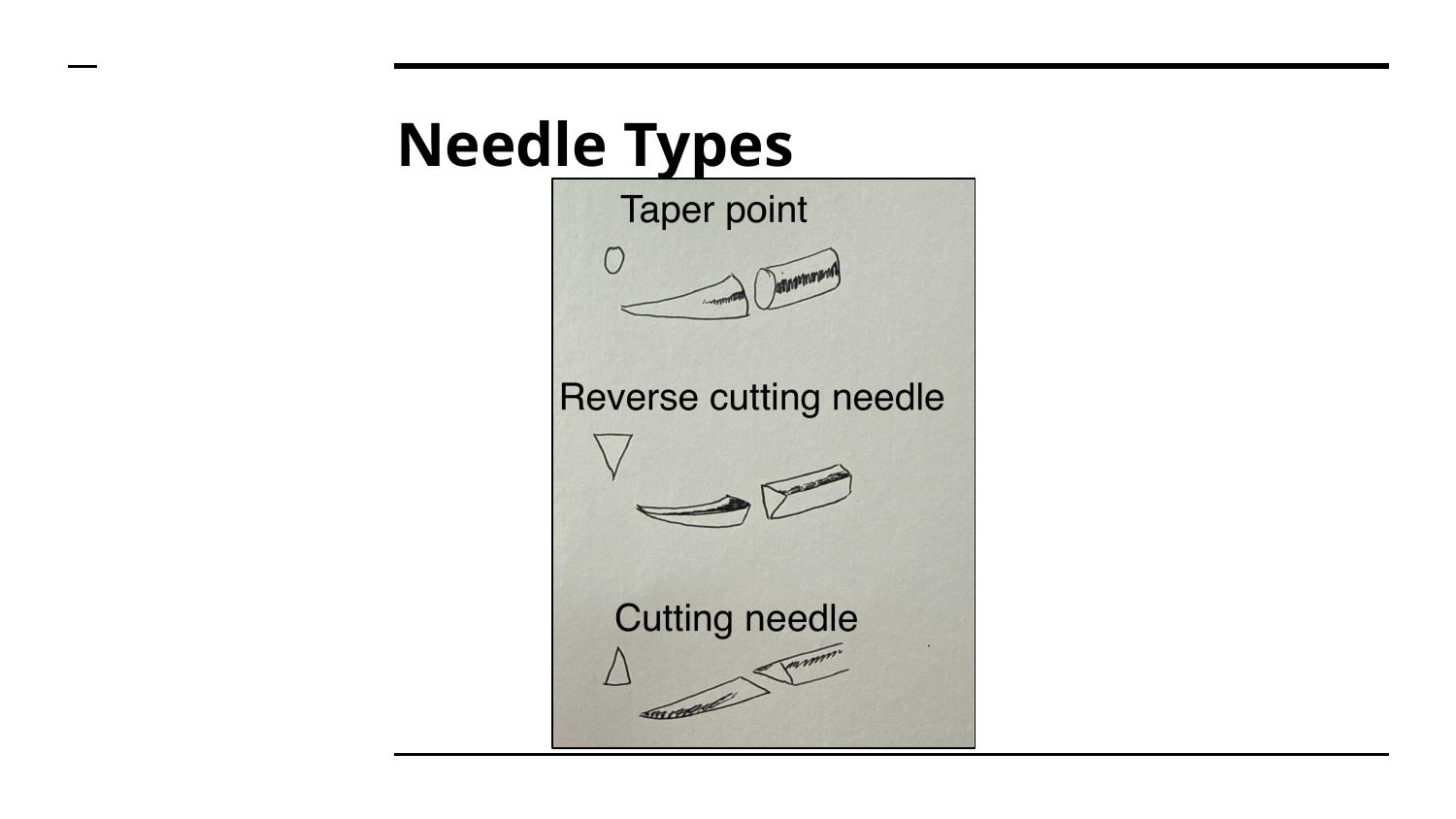

# Needle Types

## Slide 8
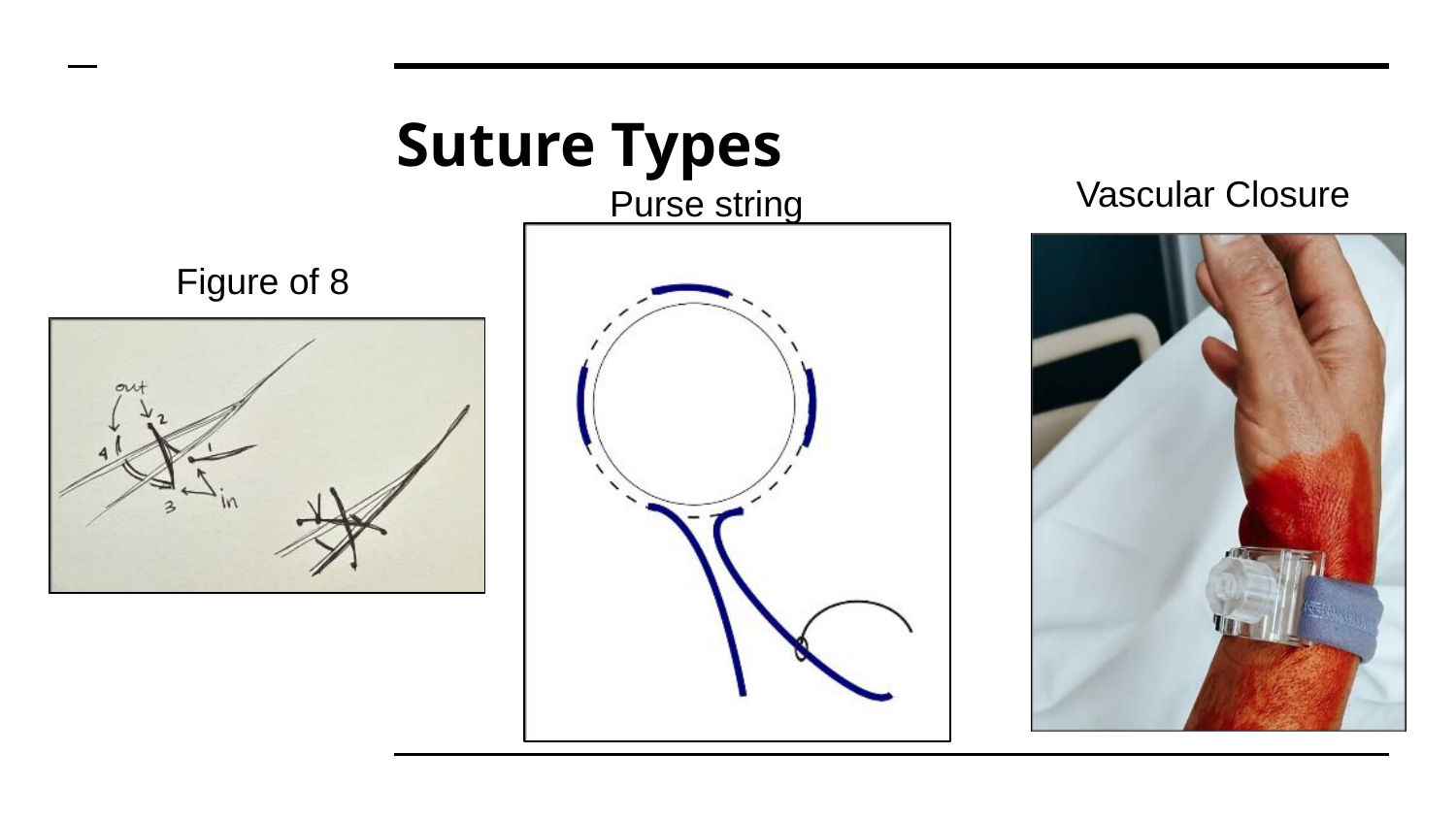

# Suture Types
Vascular Closure
Purse string
Figure of 8

## Slide 9
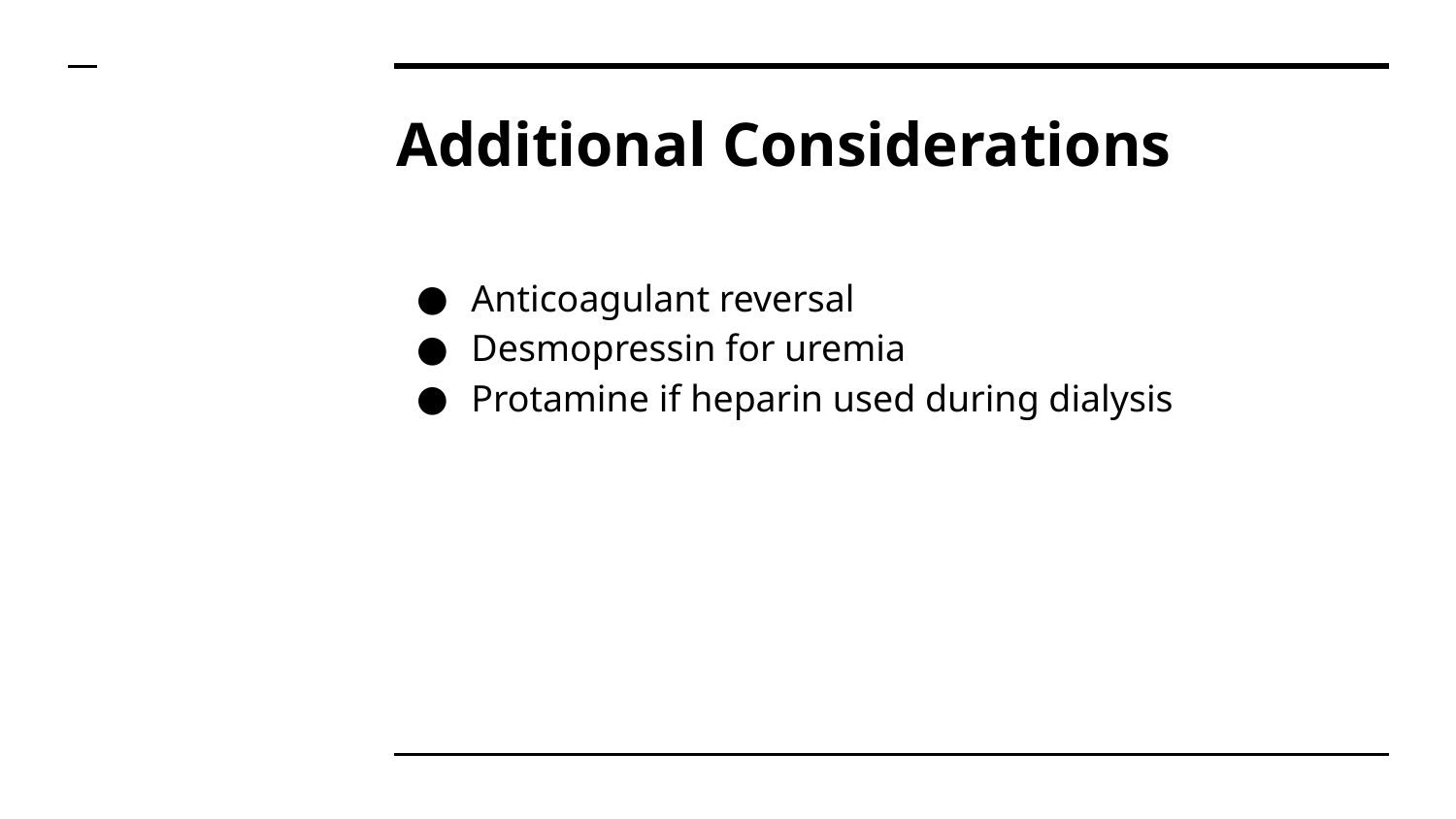

# Additional Considerations
Anticoagulant reversal
Desmopressin for uremia
Protamine if heparin used during dialysis

## Slide 10
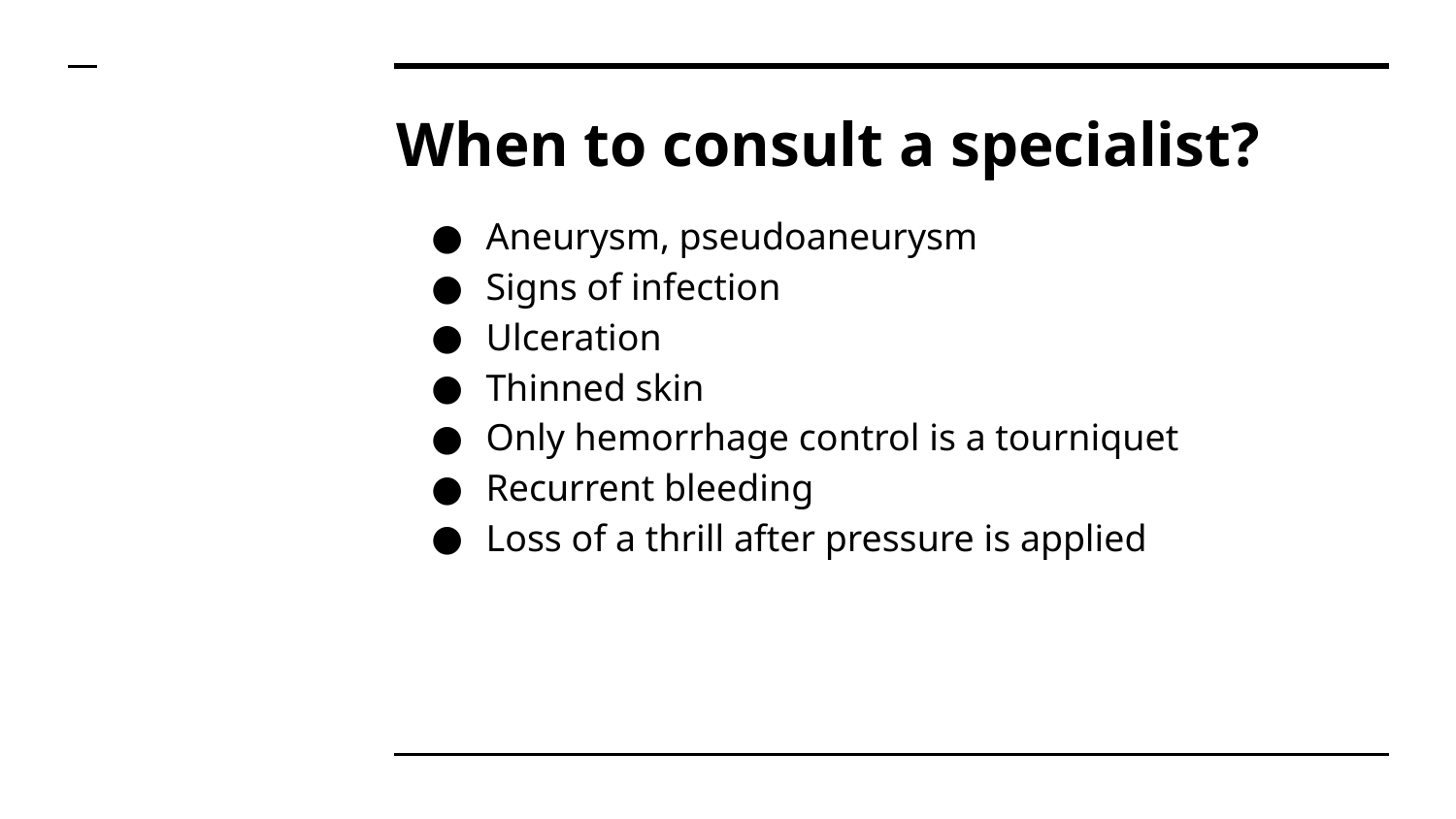

# When to consult a specialist?
Aneurysm, pseudoaneurysm
Signs of infection
Ulceration
Thinned skin
Only hemorrhage control is a tourniquet
Recurrent bleeding
Loss of a thrill after pressure is applied

## Slide 11
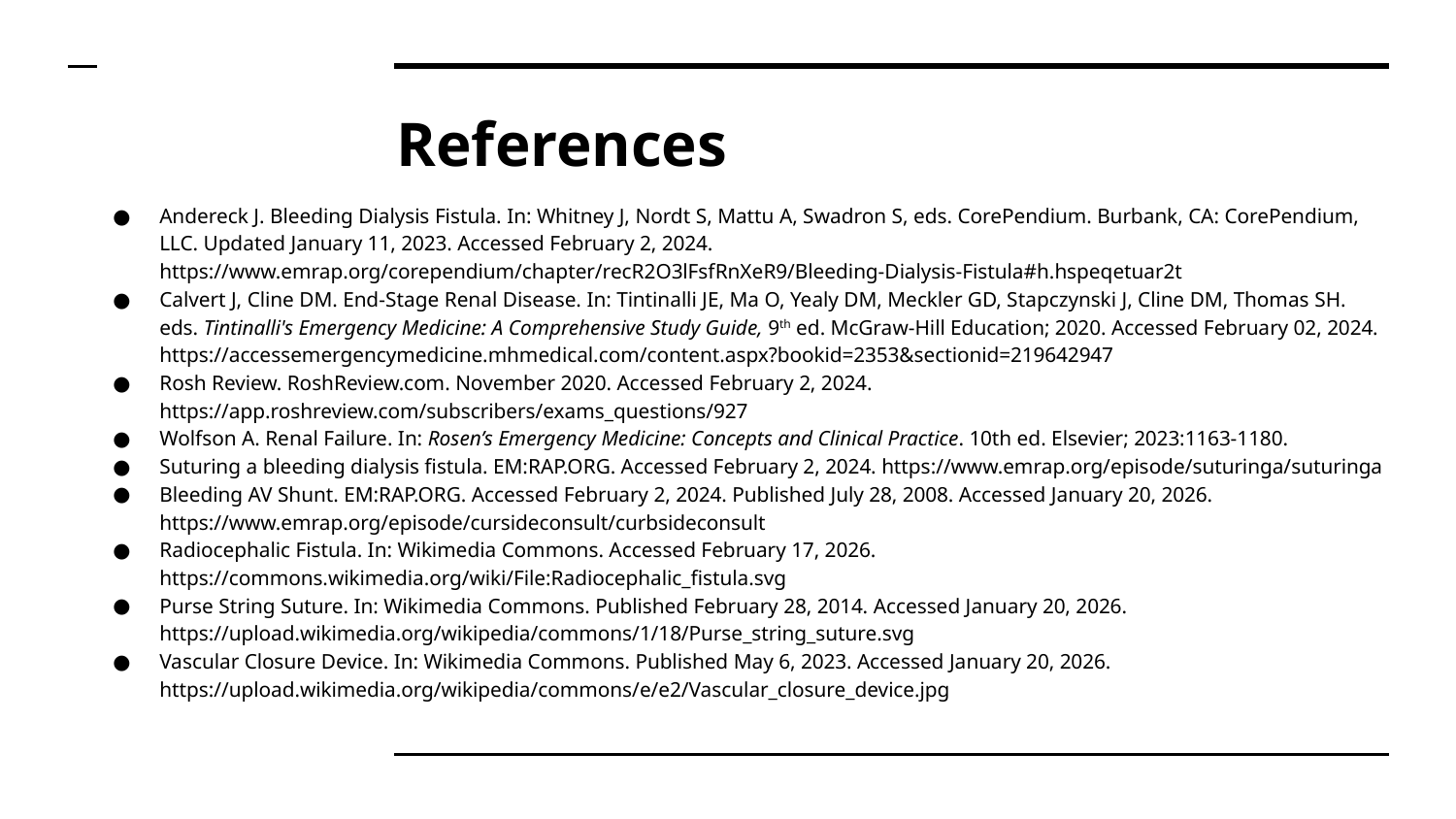

# References
Andereck J. Bleeding Dialysis Fistula. In: Whitney J, Nordt S, Mattu A, Swadron S, eds. CorePendium. Burbank, CA: CorePendium, LLC. Updated January 11, 2023. Accessed February 2, 2024. https://www.emrap.org/corependium/chapter/recR2O3lFsfRnXeR9/Bleeding-Dialysis-Fistula#h.hspeqetuar2t
Calvert J, Cline DM. End-Stage Renal Disease. In: Tintinalli JE, Ma O, Yealy DM, Meckler GD, Stapczynski J, Cline DM, Thomas SH. eds. Tintinalli's Emergency Medicine: A Comprehensive Study Guide, 9th ed. McGraw-Hill Education; 2020. Accessed February 02, 2024. https://accessemergencymedicine.mhmedical.com/content.aspx?bookid=2353&sectionid=219642947
Rosh Review. RoshReview.com. November 2020. Accessed February 2, 2024. https://app.roshreview.com/subscribers/exams_questions/927
Wolfson A. Renal Failure. In: Rosen’s Emergency Medicine: Concepts and Clinical Practice. 10th ed. Elsevier; 2023:1163-1180.
Suturing a bleeding dialysis fistula. EM:RAP.ORG. Accessed February 2, 2024. https://www.emrap.org/episode/suturinga/suturinga
Bleeding AV Shunt. EM:RAP.ORG. Accessed February 2, 2024. Published July 28, 2008. Accessed January 20, 2026. https://www.emrap.org/episode/cursideconsult/curbsideconsult
Radiocephalic Fistula. In: Wikimedia Commons. Accessed February 17, 2026. https://commons.wikimedia.org/wiki/File:Radiocephalic_fistula.svg
Purse String Suture. In: Wikimedia Commons. Published February 28, 2014. Accessed January 20, 2026. https://upload.wikimedia.org/wikipedia/commons/1/18/Purse_string_suture.svg
Vascular Closure Device. In: Wikimedia Commons. Published May 6, 2023. Accessed January 20, 2026. https://upload.wikimedia.org/wikipedia/commons/e/e2/Vascular_closure_device.jpg
